# Shrimp miR-965 induced the human melanoma stem-like cell apoptosis and inhibited their stemness by disrupting the MCL-1-ER stress-XBP1 feedback loop in a cross-species manner

**DOI:** 10.1186/s13287-020-01734-3

**Published:** 2020-06-25

**Authors:** Wenlin Wu, Chenxi Xu, Xiaobo Zhang, An Yu, Le Shu

**Affiliations:** 1 College of Oceanology and Food Science, Quanzhou Normal University, Quanzhou, 36200 Fujian Province People’s Republic of China; 2 College of Life Science, Zhejiang University, Hangzhou, 310058 Zhejiang Province People’s Republic of China; 3 Huffington Centre on Aging, Baylor College of Medicine, Houston, TX 77030 USA

**Keywords:** Melanoma stem-like cells, Myeloid cell leukemia sequence 1 (MCL-1), Shrimp miR-965, X-box-binding protein 1 (XBP1), Cross-species gene expression regulation

## Abstract

**Background:**

Melanoma is a type of aggressive skin cancer with a poor survival rate. The resistance to conventional therapy of this disease is, at least in part, attributed to its cancer stem cell population. However, the mechanism of survival and stemness maintenance of cancer stem cells remains to be investigated.

**Methods:**

Tumorsphere formation assay was used to study the stem-like property of melanoma stem-like cells (MSLC). Chromatin immunoprecipitation (ChIP), promoter luciferase reporter assay were included for exploring the role of MCL-1 in MSLC and electrophoretic mobility shift assay were used to evaluate the interaction between shrimp miR-965 and human Ago2 protein. Melanoma xenograft nude mice were used to study the inhibition of tumor development.

**Results:**

In the present study, our results showed that myeloid cell leukemia sequence 1 (MCL-1) knocking down induced ER stress and apoptosis, and the expression reduction of stemness associated genes in MSLC, which implied a significant role of MCL-1 in MSLC. Further study indicated that ER stress agonist (tunicamycin) treatment in MSLC results in the translocation of XBP1, an ER stress sensor, into the nucleus to induce MCL-1 expression through direct binding to the − 313- to − 308-bp region of MCL-1 promoter. In addition, we found that a shrimp-derived miRNA (shrimp miR-965) could interact with the human Ago2 protein and suppressed the human MCL-1 expression by binding to the 3′ UTR of MCL-1 mRNA, thereby inhibiting the MSLC proliferation and stemness in vitro and in vivo in a cross-species manner.

**Conclusion:**

In conclusion, we identified an important role of MCL-1-ER stress-XBP1 feedback loop in the stemness and survival maintenance of MSLC, and shrimp miR-965, a natural food derived miRNA, could regulate MSLC stemness and survival by targeting MCL-1 and disrupting the balance of MCL-1-ER stress-XBP1 feedback loop. In conclusion, this study indicated an important mechanism of the regulation of MSLC stemness and survival, otherwise it also demonstrated the significance of cross-species-derived miRNA as promising natural drugs in melanoma therapy.

## Introduction

Melanoma is a type of aggressive cancer featuring high mortality. Despite the fact that melanoma only explains 10% of skin cancers, it is accountable for 80% of skin cancer deaths [1, 2]. Chemotherapy and target therapies are available treatments for melanoma, which has greatly improved the 5-year survival rate of patients. However, resistance to therapeutic agents presented in cancers including melanoma raised new challenges to the further improvement of cancer therapy [3]. Accumulating investigations have revealed a specific subpopulation of cancer cells, termed cancer stem cells (CSCs), which is a critical contributor to tumor resistance to conventional chemotherapy and radiotherapy [4–6]. Featured with metabolic adaptation, CSCs are considered to possess slower cycling rate than differentiated cancer cells; they are able to re-enter into the cell cycle after radiotherapy or chemotherapy, while most differentiated cancer cells die or undergo cell cycle arrest [7, 8]. Strategies targeting cancer stem cells have been explored including intervention of signaling pathways involved in CSC self-renew or self-maintenance [9]. Myeloid cell leukemia sequence 1(MCL-1) is an anti-apoptotic protein, which belongs to BCL-2 protein family and is primarily located in the mitochondria, in which it inserts into the mitochondrial membrane via a hydrophobic tail and is implicated in the ER-mitochondria signaling transmission [10–12]. The anti-apoptotic function of MCL-1 is essential to cell survival and homeostasis [11]. The BCL-2 family proteins can be antiapoptotic or proapoptotic. The antiapoptotic BCL-2 proteins include MCL-1, BCL-2 (BCL2 apoptosis regulator), BCL-W (BCL2 like 2), and BCL-XL (BCL2-like 1), while such as BAK (BCL-2 homologous antagonist killer) and BAX (BCL-2 associated X protein) were pro-apoptotic BCL-2 proteins. The interactions among these proteins determine the fate of cells [13–15]. Overexpression of MCL-1 have been reported in a wide range of human tumors, such as melanoma, non-small cell lung cancer, breast cancer, and ovarian cancer [16]. Given the anti-apoptotic property of MCL-1, MCL-1 has been shown to be both an intrinsic and acquired resistance factor that impairs the potency of various antitumor agents, and studies have suggested the utility of cancer therapy targeting MCL-1 based on the anti-apoptotic property of MCL-1 [13]. However, as a therapeutic target of cancer, the relationship between MCL-1 and CSC is poorly explored.

miRNAs are the non-coding single-stranded RNA of ~ 22 nucleotides which are derived from the endogenously produced pre-miRNA (precursor) of 75–80 nucleotides with a hairpin (stem-loop) structure [17]. They regulate their target genes by binding to the 3′ UTR region of the target mRNAs, which causes translational repression [17]. In recent years, miRNAs have been proven to have a profound impact on many patho-physiologic processes including proliferation, apoptosis, and stress response. As miRNAs have the ability to target numerous mRNAs by binding to their seed sequences, single small RNA molecules may operate highly complex regulatory networks and regulate the expression of genes in different species [18]. However, the cross-species regulations of miRNAs have not been extensively investigated. Therefore, in this study, we explored the role of shrimp miR-965 as a natural drug for melanoma therapy by disrupting the MCL-1-ER stress-XBP1 feedback loop in melanoma stem-like cells (MSLC).

## Materials and methods

### Melanoma stem-like cells culture

The melanoma stem-like cells were a gift from Dr. Zhang’s lab. The sorting of melanoma stem-like cells was conducted using an ALDEFLUORTM kit (Cyagen Biosciences Inc., USA) for the detection of melanoma stem cell marker, aldehyde dehydrogenase 1 (ALDH1) [19]. Briefly, MDA-MB-435 cells were suspended in ALDEFLUOR assay buffer containing ALDH1 fluorescent substrate BODIPY-aminoacetate (BAAA, 1 μM) and incubated for 40 min at 37 °C. After incubation, the cells were centrifuged at 250×*g* for 5 min followed by removing the supernatant; the cell pellet was resuspended in 0.5 mL of ALDEFLUOR assay buffer and stored at 4 °C for fluorescence-activated cell sorting (FACS). The ALDH1-positive cells were termed as melanoma stem-like cells (MSLC), and the rest were noted as melanoma non-stem-like cells (non-MSLC). The sorted melanoma stem-like cells were maintained immediately in DMEM/F-12 medium supplemented with 20 ng/mL epidermal growth factor (Beyotime, China), 10 ng/mL basic fibroblast growth factor (Beyotime, China), 5 μg/mL of insulin (Beyotime, China), and 2% of B27 (Sigma, USA).

### Tumorsphere formation assay

Tumorsphere formation assay was conducted under non-adherent and serum-free conditions. To perform the tumorsphere formation assay, the cells were firstly transfected with indicated siRNAs/miRNAs for 6 h. After that, the cells were counted and seeded in 6-well ultralow adherent cell culture plate, and the number of cells was 5000 in each well. The cells were cultured in DMEM/F-12 medium supplemented with 20 ng/mL epidermal growth factor (Beyotime, China), 10 ng/mL basic fibroblast growth factor (Beyotime, China), 5 μg/mL of insulin (Beyotime, China), and 2% of B27 (Sigma, USA). Seven days after seeding, the tumorspheres were (each tumorsphere should contain at least 5 cells) detected and analyzed.

### Quantification of mRNA with real-time PCR

Total RNAs were isolated using a commercial RNA isolation kit (Ambion, USA) according to the manufacturer’s instructions. Reverse transcription was performed with a reverse transcription kit (Toyobo, Japan) thus converting mRNA to cDNA. The real-time PCR reaction consisted of 0.5 μL of cDNA and 5 μL of 2× Premix Ex Taq (Takara, Japan); 0.5 μL each of primers was conducted at 95 °C for 10 min, followed by 40 cycles at 95 °C for 15 s and 60 °C for 30 s. Transcripts of the genes of interest were detected by real-time RT-PCR using gene-specific primers. GAPDH mRNA was used for normalization. The primers were as follows:

Oct-3/4: 5′-GAGCAAAACCCGGAGGAGT-3′ and 5′-TTCTCTTTCGGGCCTGCAC-3′

Nanog: 5′-GCTTGCCTTGCTTTGAAGCA-3′ and 5′-TTCTTGACTGGGACCTTGTC-3′

ALDH1: 5′-TTACCTGTCCTACTCACCGA-3′ and 5′-CTCCTTATCTCCTTCTTCTACCT-3′

ABCG2: 5′-GGCCTCAGGAAGACTTATGT-3′ and 5′-AAGGAGGTGGTGTAGCTGAT-3′

GAPDH: 5′-GGTATCGTGGAAGGACTCATGAC-3′ and 5′-ATGCCAGTGAGCTTCCCGTTCAG-3′

MCL-1: 5′-AAGCCAATGGGCAGGTCT-3′ and 5′-TGTCCAGTTTCCGAAGCAT-3′

Chop: 5′-CGACAGAGCCAGAATAACAGC-3′ and 5′-AAGGTGAAAGGCAGGGACTC-3′

ATF4: 5′-TGAAGGAGTTCGACTTGGATGCC-3′ and 5′-CAGAAGGTCATCTGGCATGGTTTC-3′

ATF3: 5′-CTCCTGGGTCACTGGTGTT-3′ and 5′-TCTGAGCCTTCAGTTCAGCA-3′

XBP1s (spliced XBP1): 5′-GAGTCCGCAGCAGGTG-3′ and 5′-TCCTTCTGGGTAGACCTCTGGGAG-3′

EDEM1: 5′-CCAGATGGTTGGCTTGATT-3′ and 5′-AGAGCTGGACAGAAACTTCG-3′

Herp: 5′-CTTGGAGCTGAGTGGCGAC-3′ and 5′-CAATGTCCAGGAGAGGCAATC-3′

### Western blot

Cell lysates were separated using 12% SDS-PAGE and then transferred to the PVDF membrane. The membrane was blocked with triethanolamine-buffered saline (TBS) solution containing 5% skim milk. Subsequently, the membrane was incubated overnight with the antibody of interest, followed by incubation with the HRP-conjugated secondary antibody (Roche, Switzerland) for 2 h at room temperature. After a rinse, the membrane was detected visualized using an enhanced-chemiluminescence (ECL) detection system (Beyotime, China).

### RNA interference

The siRNAs or miRNAs were transfected into cells using the Lipofectamine transfection reagent (Life Technology, USA) according to the manufacturer’s manual. The siRNAs and miRNAs used in the experiments were as follows:

MCL-1-siRNA: 5′-GCAGGAUUGUGACUCUCAUTT-3′

XBP1-siRNA: 5′-TGCCAATGAACTCTTTCCCTT-3′

shrimp miR-965: 5′-UAAGCGUAUGGCUUUUCCCCUC-3′

Twenty-four hours after transfection, cells were collected for further study. The siRNAs and miRNAs were synthesized by GenePharma Co., Ltd. (Shanghai, China).

### Truncation assay

To conduct truncation assay, MSLC were co-transfected with pGL3 plasmid fused with different truncated or mutant MCL-1 promoters including − 1000~+ 100 bp, − 550~+ 100 bp, and − 400~+ 100 bp, as well as the − 1000~+ 100 bp with a mutant sequence within − 313~− 124 bp; these truncated promoters were subsequently notated as pGL3-MCL-1 (− 1000), pGL3-MCL-1 (− 550), pGL3-MCL-1 (− 400), pGL3-MCL-1 (− 200), pGL3-MCL-1 (− 313 mut), respectively, and pcDNA3.1-XBP1s or pcDNA3.1-control as well as 20 ng of Renilla luciferase followed by assessing luciferase activity of the treated cells.

### Promoter luciferase reporter assay

Cells were transfected with 200 ng of recombinant pGL3 plasmid containing truncated human MCL-1 promoters (or mutant MCL-1 promoter) and 20 ng of Renilla luciferase (pRL-TK) vector (Promega, USA) as well as pcDNA3.1-XBP1s or pcDNA3.1-control. Twenty-four hours later, luciferase activity was quantified according to the manufacturer’s protocol (Promega, USA).

### Chromatin immunoprecipitation

Chromatin immunoprecipitation (ChIP) was performed using the Simple ChIP Plus Enzymatic Chromatin IP Kit (Magnetic Beads) (Cell Signaling Technology, USA) following the manufacturer’s instructions. For each immunoprecipitation reaction, 5 μg of human XBP1s antibody (Cell Signaling Technology, USA) was used for every 5 μg of pre-cleared chromatin.

### PmirGLO dual-luciferase reporter assay

To evaluate the interaction between shrimp miR-965 and MCL-1, the MCL-1 mutant 3′ UTR (5′-TTCTGTTTGTCTATGCGAACTCTCA-3′) which is mutated in the sites matched the seed region of shrimp miR-965 and the wild-type MCL-1 3′ UTR (5′-TTCTGTTTGTCTTACGCTTCTCTCA-3′) were cloned into the pmirGLO Dual-Luciferase miRNA Target Expression Vector (Promega, USA). After that, 50 nM of shrimp miR-965 (or shrimp miR-965-scrambled) was co-transfected with 100 ng of the pmirGLO- MCL-1 3′ UTR plasmid or the pmirGLO-mutant MCL-1 3′ UTR plasmid into cells using Lipofectamine 2000. At 24 h after co-transfection, the Firefly luciferase and Renilla luciferase activities were evaluated using the dual-luciferase reporter assay system (Promega, USA) according to the manufacturer’s protocol.

### Electrophoretic mobility shift assay

To perform the electrophoretic mobility shift assay (EMSA) assay, different concentrations (12.5, 25, 50, or 100 mM) of recombinant human Ago2 were incubated with 40 mM of shrimp miR-965. After incubation in the reaction buffer (0.1 M KCl, 1 mM DTT, 1 mM MgCl2, 10 Mm HEPES, pH 7.6) for 30 min at 37 °C, the mixture was separated on a 1% agarose gel at 120 V for 0.5 h. Then the RNA bands were stained by ethidium bromide, and subsequently, the proteins were stained with Coomassie Blue.

### Tumor initiator and development in nude mice

To assess the effect of shrimp miR-965 on the tumor-initiating ability and tumor development of MSLC in vivo, 2 × 10^3^ cells scattered in 100 μL PBS solution were injected into each nude mouse (approximately 4 weeks old) from the tail vein. Three days later, either shrimp miR-965 or shrimp miR-965-scrambled was injected into the mice via the tail vein at a dosage of 80 mg/kg of body weight. Shrimp miR-965 or shrimp miR-965-scrambled was injected every 3 days over an 8-week period. All the in vivo experiments were performed in accordance with a protocol approved by the Institutional Animal Care and Use Committee (IACUC). After that, the lungs of the mice were collected at the end of the experimental period for further research.

### Northern blot analysis

Total small RNAs extracted from indicated tissues using a mirVana miRNA isolation kit (Ambion, USA) were separated by electrophoresis on a 15% polyacrylamide gel containing 7 M urea for 2 h at 5 mA, then transferred to a nylon membrane (Amersham Biosciences, UK) at 300 mA for 2 h. Subsequently, the membrane was cross-linked by UV and then prehybridized in a prehybridization solution (Roche, Switzerland) for 30 min. To detect the target miRNA, the membrane was hybridized with digoxigenin (DIG)-labeled shrimp miR-965 probe (5′-GAGGGGAAAAGCCATACGCTTA-3′) overnight. After that, the membrane was rinsed and then blocked in a blocking solution (Roche, Switzerland) for 1 h at room temperature. Lastly, the membrane was incubated with the antibody against DIG-labeled alkaline phosphate (Roche, Switzerland) for 2 h, and the target miRNA was visualized with the substrate BCIP/NBT solution (Roche, Switzerland).

### Caspase 3/7 activity detection

To assess the activities of the caspase 3/7, cells at a density of 1 × 10^4^/well were plated in a 96-well plate and transfected with siRNA or miRNA. Thirty-six hours later, 100 μL of caspase-Glo 3/7 reagent (Promega, USA) was added to each well. After incubation in the dark at room temperature for 30 min, the luminescence of cells was measured. This assay was conducted according to the caspase-Glo 3/7 kit (Promega, USA) manufacturer’s protocol.

Apoptosis detection with annexin V.

### Evaluation of cell apoptosis with annexin V

The apoptosis of cells was detected with the FITC annexin V apoptosis detection kit I (Becton, Dickinson and Company, USA). Cells were collected and rinsed with cold phosphate-buffered saline (PBS) and then resuspended in 1× annexin binding buffer at 1 × 10^6^ cells/mL. Subsequently, after that, 5 μL of Alexa Fluor488 Annexin V and 0.1 μg of PI (propidium iodide) were mixed with the cells. After incubation at room temperature for 15 min in the dark, the sample was added with 400 μL of 1× annexin binding buffer and then subjected to analysis with a flow cytometer at an excitation of 575 nm.

### MTS assay

Cell viability and proliferation analysis were conducted with MTS [3-(4, 5-dimethylthiazol-2-yl)-5-(3-carboxymethoxyphenyl)-2-(4-sulfophenyl)-2H-tetrazolium, inner salt] assays (Promega, USA). Cells seeded onto a 96-well plate (containing 100 μL of culture medium) were added with 20 μL of MTS reagent. Then, the plate was subjected to incubation for 1.5 h at 37 °C in a humidified incubator containing 5% CO_2_. The absorbance at 450 nm was recorded. Cell proliferation rate analysis was performed via calculating cell viability of time-course assays.

### Shrimp miR-965 mediated consecutive tumorsphere formation inhibition

MSLC transfected with miRNA-965 (or shrimp miR-965-scrambled) for 6 h were seeded in 6-well ultralow adherent cell culture plate, and the number of cells was 5000 in each well. Five days later, the sphere number in each well was detected (first generation). After that, tumorspheres were re-suspended into scattered single cells and reseeded into 6-well ultralow adherent cell culture plate (cell number in each well was kept in 5000). Five days later, the sphere number in each well was detected (second generation). Repeated the re-suspend and seeded process and detected the sphere number 5 days after seeding (third generation).

### H&E staining

Bring the slides from the − 80 °C freezer to room temperature. Incubate the slides with hematoxylin solution in a staining jar for 10 min to stain the nuclei. Transfer the slides to a staining jar with running water (tap water is fine) till the water is clear. Transfer the slides to a staining jar with eosin solution for 3 min. Successively transfer the slides into staining jars with 70% ethanol for 20 s, 90% ethanol for 20 s, 100% ethanol for 1 min, and xylene for 3 min. Take out slides from xylene and place the slides in a fume hood till the slides are dry. Mount the slides with xylene-based mounting media and cover with cover slides. Clips are used to press the slides to squeeze bubbles. Store the slides at room temperature. Hematoxylin and eosin-stained images were captured with a 14.0-MP digital microscope camera which is attached via a c-mount to the side port of a Leica DMI 6000B microscope.

### Statistical analysis

All data were presented as mean ± standard deviation. Numerical data were processed using one-way analysis of variation (ANOVA), and Student’s *t* test was employed to assess the significant difference. All assays were biologically repeated for three times.

## Results

### MCL-1 is upregulated in MSLC

**Fig. 1:**
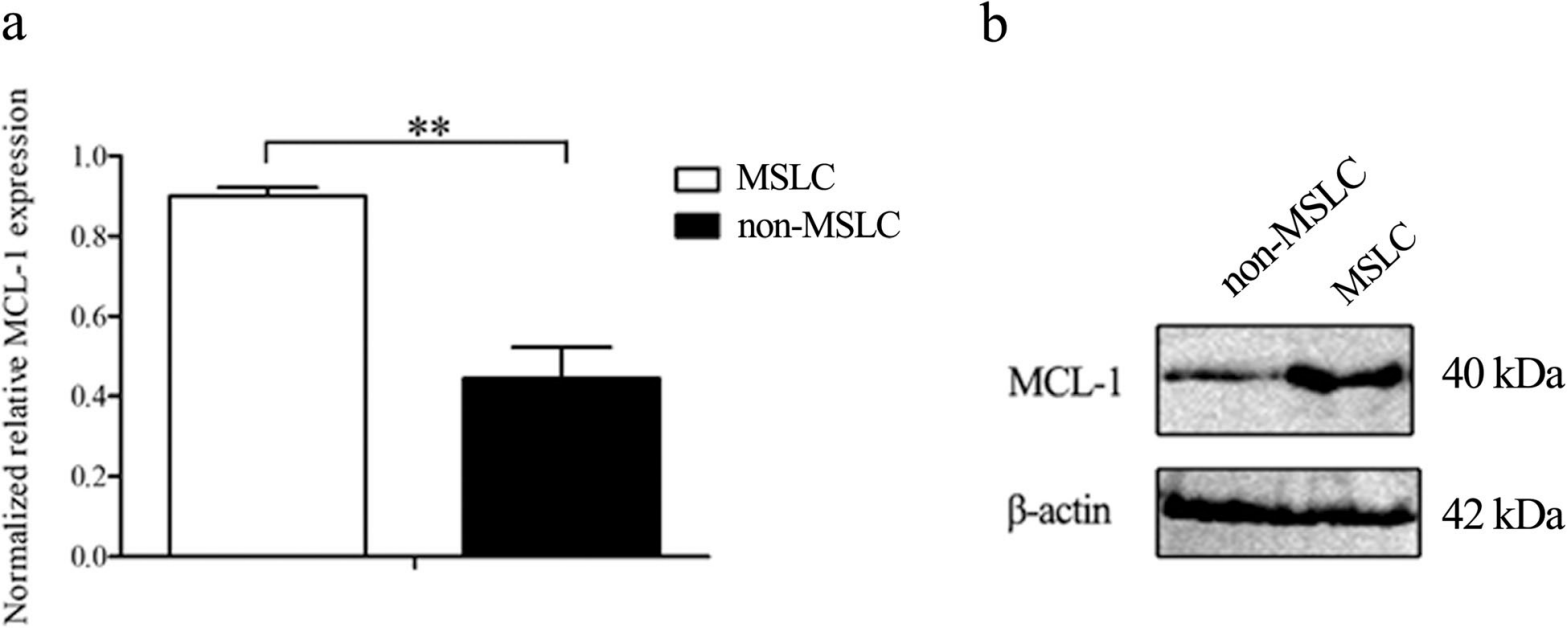
MCL-1 is upregulated in melanoma stem-like cells (MSLC). **a** The mRNA level of MCL-1 in MSLC and melanoma non-stem-like cells (non-MSLC) was detected by quantitative real-time PCR (**p < 0.01). **b** The MCL-1 protein in MSLC and non-MSLC was detected by Western blot

MCL-1 is an anti-apoptotic Bcl-2 family member that is overexpressed in many types of tumors. MCL-1 inhibits cell death by binding to proapoptoptic Bcl-2 family proteins thus suppressing caspase activation [20]. To reveal the role of MCL-1 in the progression of MSLC, we firstly characterize the expression of MCL-1 in MSLC. The data of quantitative real-time PCR and Western blots showed that MCL-1 was significantly upregulated in the MSLC compared with that in non-MSLC (Fig. 1a, b), indicating that MCL-1 may play an important role in MSLC.

### MCL-1 is necessary for the stemness and survival of MSLC

**Fig. 2:**
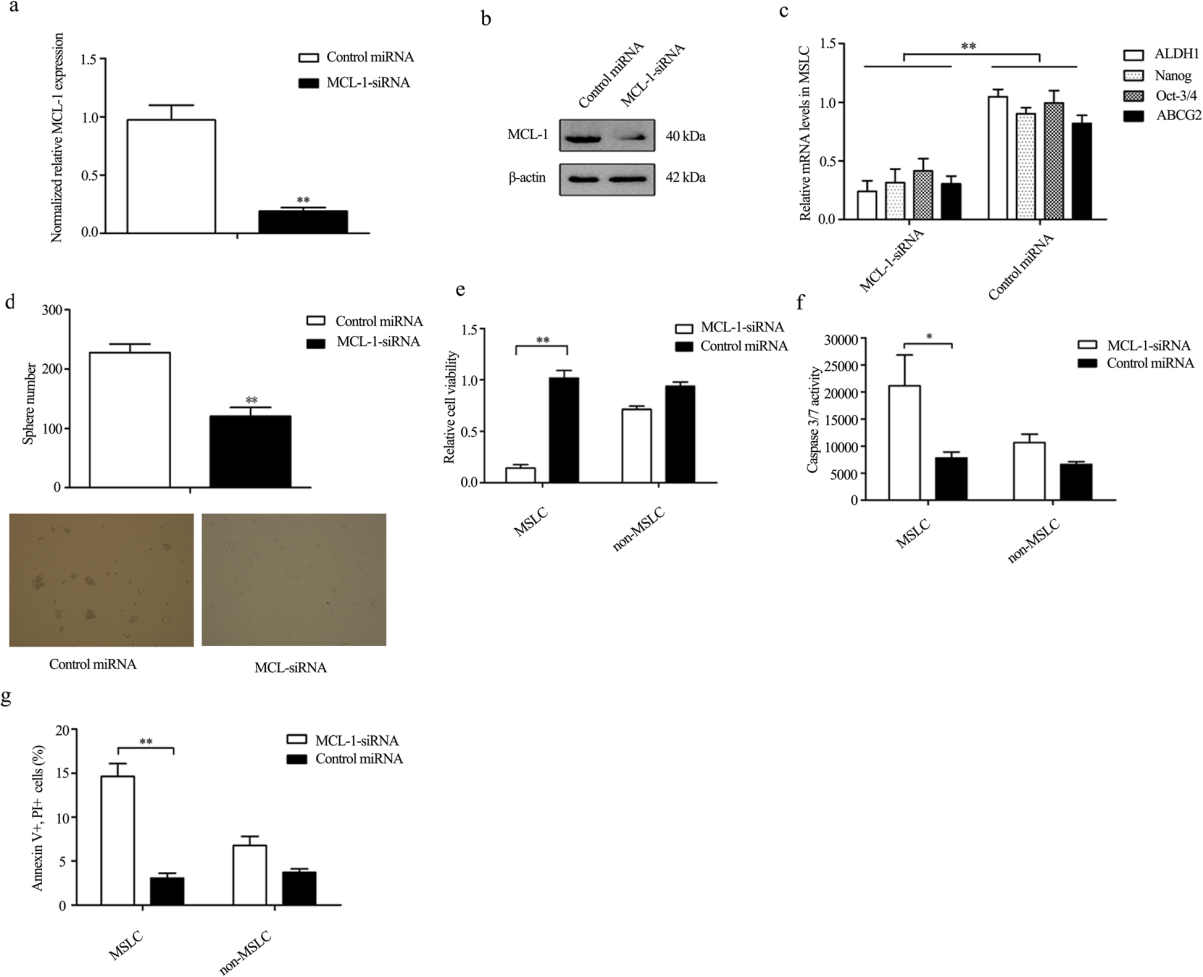
MCL-1 is required for the MSLC. **a** The MCL-1 mRNA level in MSLC transfected with MCL-1-siRNA. At 24 h after transfection, the mRNA level of the cells was evaluated using quantitative real-time PCR (**p < 0.01). The MCL-1-siRNA-scrambled was used as a control. **b** The knockdown of MCL-1 expression at the protein level in MSLC by MCL-1-siRNA. **c** MCL-1 silencing inhibited the expression of stemness-associated genes in MSLC. At 24 h after transfection of MCL-1-siRNA in MSLC, quantitative real-time PCR was used to evaluate the expression levels of stemness associated genes (**p < 0.01). **d** MCL-1 knockdown reduced the tumorsphere formation capacity of MSLC. The expression of MCL-1 was silenced by siRNA in MSLC. Seven days after cell seeding, the tumorsphere numbers of MSLC were examined. Control miRNA was used as a control. **e** The impact of MCL-1 silencing on the MSLC and non-MSLC viability (**p < 0.01). **f** Knockdown of MCL-1 induced MSLC apoptosis but not in non-MSLC. The MSLC and non-MSLC were treated with MCL-1-siRNA. Twenty-four hours later, apoptosis was examined with the caspase 3/7 activity assay (*p < 0.05). **g** The detection of apoptosis using annexin V assay. Apoptosis of MSLC and non-MSLC was evaluated by flow cytometry at 24 h after the transfection of MCL-1-siRNA (**p < 0.01)

To further explore the role of MCL-1 in the MSLC. The impact of MCL-1 on the stemness of MSLC was evaluated; quantitative real-time PCR and Western blot results showed that MCL-1 expression was silenced by MCL-1 siRNA in MSLC (Fig. 2a, b). Then, the expression levels of stemness-associated genes in MCL-1-silenced MSLC were examined to evaluate the effects of MCL-1 silencing on the stemness of MSLC. The results showed that the CSC-associated genes (*Oct-3/4*, *Nanog*, *ALDH1*, and *ABCG2*) [21–24] were significantly downregulated when MCL-1 was silenced with siRNA (Fig. 2c), indicating the involvement of MCL-1 in the stemness of the MSLC. The investigations of tumorsphere formation assays revealed that MSLC treated with MCL-siRNA showed less tumorspheres in comparison with those in the control group, which indicated the sphere-forming capacity of MSLC was significantly suppressed when the MCL-1 expression was suppressed (Fig. 2d). These results implied that MCL-1 was necessary for the stemness of MSLC.

Based on the anti-apoptotic function of MCL-1, MTS assays were conducted to investigate the effects of MCL-1 on the growth of MSLC. Our results indicated that the silence of MCL-1 led to a significant decrease of MSLC viability (Fig. 2e) and a significant increase of Caspase 3/7 activity (Fig. 2f), showing that the MCL-1 knockdown could induce MSLC apoptosis. The data from annexin V assays further revealed that following the transfection of MCL1-siRNA into cells, the percentage of apoptotic cells (PI and annexin V-positive) was significantly increased in MSLC (Fig. 2g). Yet, the downregulation of MCL-1 had no comparable impact on non-MSLC.

### The endoplasmic reticulum stress might be responsible for the apoptosis and stemness reduction mediated by MCL-1 inhibition

**Fig. 3:**
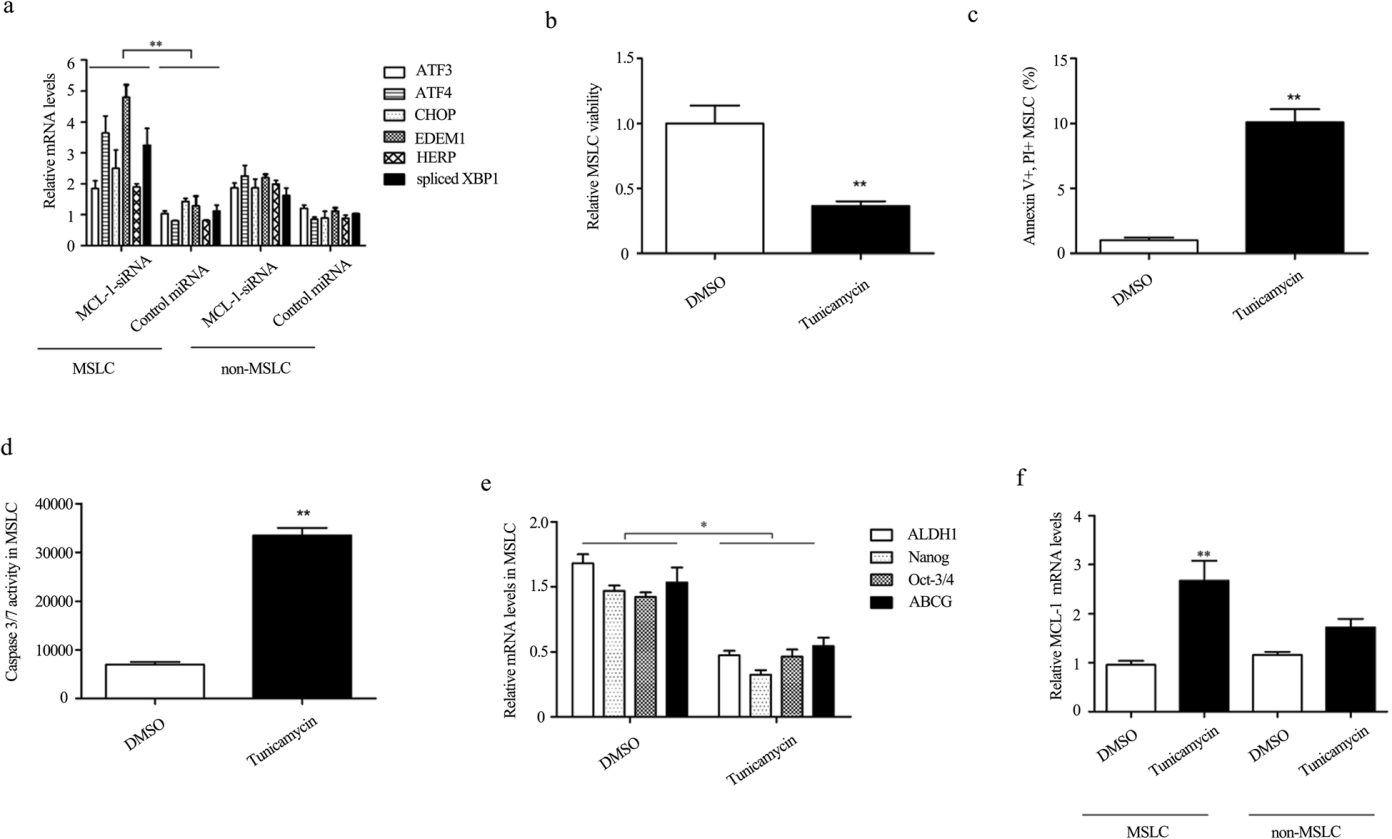
The endoplasmic reticulum stress might be responsible for the apoptosis and stemness reduction mediated by MCL-1 in MSLC. **a** MCL-1 silencing upregulated the expression of ER stress-associated genes in MSLC but not in non-MSLC. At 24 h after transfection of MCL-1-siRNA in MSLC and non-MSLC, quantitative real-time PCR was used to evaluate the expression levels of ER stress-associated genes (**p < 0.01). The MCL-1-siRNA-scrambled was used as a control (control miRNA). **b**–**d** MSLC was treated with ER stress agonist tunicamycin (3 μg/mL) for 24 h followed by evaluating the cell viability and apoptosis using MTS assay, annexin V assay, and caspase 3/7 activity detection, respectively. **e** Influence of tunicamycin on CSC-associated gene in MSLC. Cells were treated with 3 μg/mL of tunicamycin for 24 h, and then the CSC-associated genes in MSLC were assessed. **f** MCL-1 was induced by tunicamycin in MSLC but not in non-MSLC. The cells were subjected to tunicamycin (3 μg/mL) treatment for 24 h followed by detecting the MCL-1 mRNA content

Previous studies have shown that the upregulation of MCL-1 is a major adaptive mechanism of melanoma cells to endoplasmic reticulum stress (ER stress) [25]. Therefore, in the present study, we explored the effects of MCL-1 on ER stress in MSLC. As shown in Fig. 3a, silencing of MCL-1 induced the mRNA expression of ER stress-associated genes, including ATF3, ATF4, CHOP, EDEM1, HERP, and spliced XBP1, indicating MCL-1 was involved in ER stress response. Considering the ER stress was a great challenge for cell homeostasis, and several reports had shown a significant impact of ER stress on stemness and apoptosis of CSC [26, 27], we next investigated whether the ER stress induced by MCL-1 knockdown was responsible for the cell apoptosis and stemness inhibition. The results revealed that induction of ER stress with tunicamycin (ER stress agonist) led to the decrease of the relative cell viability in MSLC (Fig. 3b) when compared with the treatment of DMSO. Otherwise, MSLC apoptosis was induced when the cells were treated with tunicamycin (Fig. 3c, d). Meanwhile, the CSC-associated genes were significantly downregulated when the MSLC were treated with tunicamycin (Fig. 3e). These results manifested knocking down MCL-1 caused the ER stress in MSLC, and the induced ER stress might be responsible for apoptosis, cell viability decrease, and stemness reduction.

Interestingly, MCL-1 expression was found to increase along with the tunicamycin treatment in MSLC, yet the MCL-1 expression was not observably induced in non-MSLC, which indicated the MCL-1 was also in reverse regulated by ER stress specially in MSLC (Fig. 3f).

### Transcriptional regulation of MCL-1 by XBP1 in a feedback manner

**Fig. 4:**
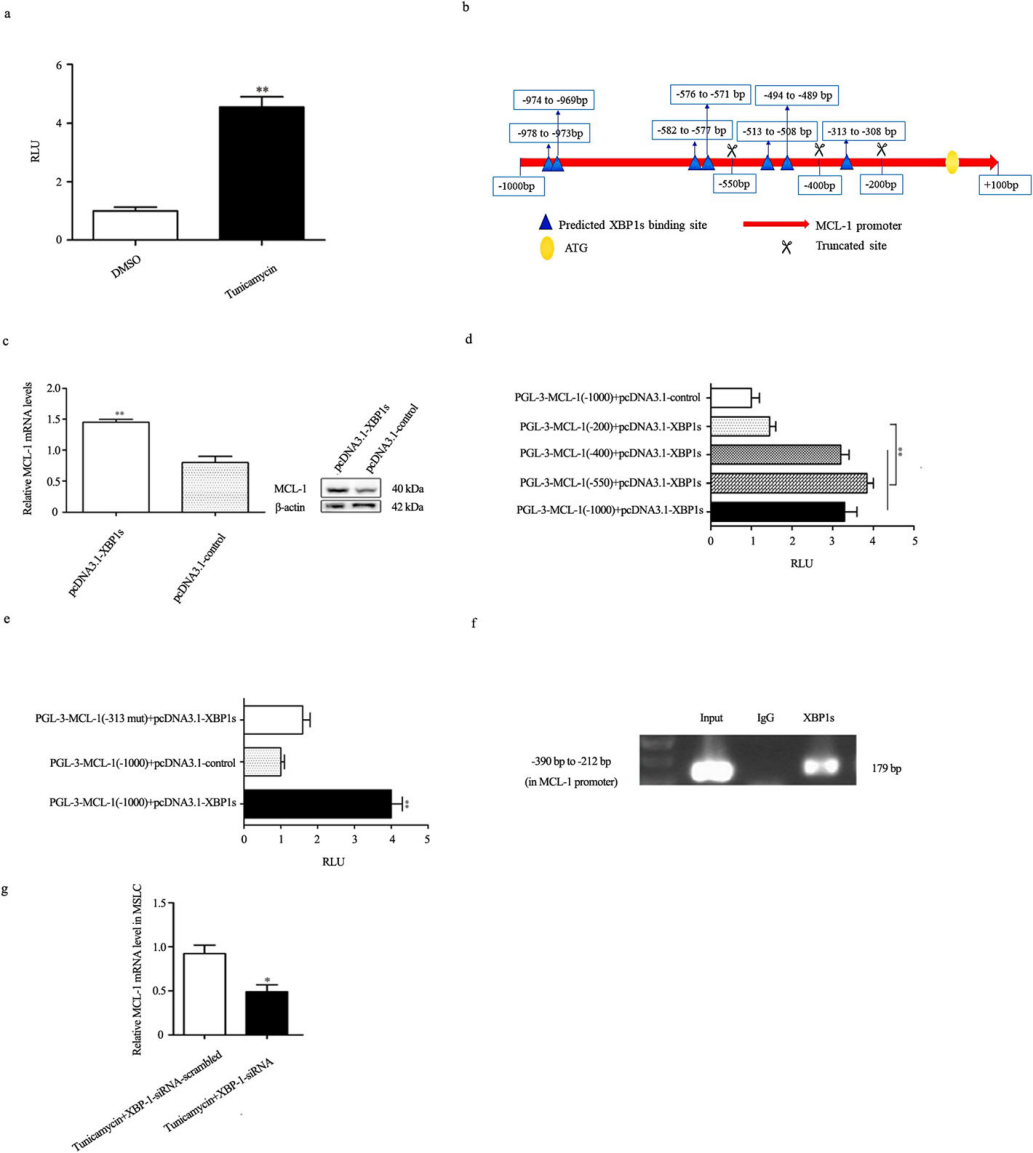
Transcriptional regulation of MCL-1 by XBP1 in a feedback manner in MSLC. **a** MCL-1 promoter was activated by tunicamycin in MSLC. MCL-1 promoter (− 1000 to + 100 bp) was inserted into the PGL3 vector to generate PGL3-MCL-1 (− 1000) and then the recombinant vector was co-transfected with the internal control pRL-TK into MSLC pre-treated with 3 μg/mL of tunicamycin followed by evaluating the fluorescence in cells. **b** Prediction of transcriptional factors that activated MCL-1 promoter during ER stress. As predicted, XBP1s was a candidate transcriptional factor that induced MCL-1 promoter activation during ER stress. **c** MSLC were transfected with pcDNA-XBP1s (or pcDNA3.1-control) followed by assessing the MCL-1 expression. **d**, **e** Identifying the XBP1s binding sites in MCL-1 promoter. MSLC were transfected with the MCL-1 wild-type promoter pGL3-MCL-1 (− 1000) (or PGL3 vectors containing truncated promoters or mutant promoter) and pcDNA3.1-XBP1s (or pcDNA3.1-control) as well as pRL-TK. Twenty-four hours later, the fluorescence of cells was evaluated. **f** Binding of XBP1s to the MCL-1 promoter was determined by chromatin immunoprecipitation (ChIP) in MSLC. ChIP assays were performed using XBP1s antibody and non-immune IgG. − 390 to − 212 bp in MCL-1 promoter was amplified during PCR in ChIP assay. **g** MCL-1 expression was feedback regulated by XBP1s through ER stress. MSLC were treated with XBP1s-siRNA (or XBPS-siRNA-scrambled) and tunicamycin. Twenty-four hours later, MCL-1 expression was detected

Our results revealed that MCL-1 was specifically induced by ER stress in MSLC at mRNA level (Fig. 3f), and the mRNA content accumulation might be resulted by the activation of the transcription of MCL-1. So next, we tested whether MCL-1 transcription was activated under ER stress. As Fig. 4a shows, tunicamycin treatment enhanced the transcription of MCL-1, implying MCL-1 transcription increase accounted for the MCL-1 mRNA upregulation in response to tunicamycin treatment in MSLC.

To further explore the mechanism of ER stress-inducing the MCL-1 transcription, potential transcriptional factors that might bind to MCL-1 promoter and increased the MCL-1 transcription under ER stress were predicted in the ALGGEN PROMO website. According to the results, XBP1, which can be spliced during ER stress to form mature transcriptional factor [28], was found to be a candidate transcriptional factor responsible for MCL-1 transcription activation (Fig. 4b).

To confirm the involvement of XBP1 in the regulation of MCL-1 expression, we firstly tested if spliced XBP1 (XBP1s) overexpression could lead to the upregulation of MCL-1. As shown in Fig. 4c, MCL-1 expression was increased at the transcript and translational levels in the condition of XBP1s overexpression, implying the XBP1 was implicated in the MCL-1 regulation.

To further evaluate whether XBP1s could induce MCL-1 transcription and map the specific binding site of XBP1s within MCL-1 promoter, consecutive length of segments of MCL-1 promoter covering different numbers of putative binding sites were cloned into pGL3-basic-based luciferase reporter constructs. The luciferase activity assay revealed a significantly increased transcriptional activity of cells with PGL3-MCL-1 (− 1000) or PGL3-MCL-1 (− 550) or PGL3-MCL-1 (− 400) or PGL3-MCL-1(-200) after pcDNA3.1-XBP1s co-transfection in comparison with cells co-transfected with pcDNA3.1-control. While shortening the promoter into − 200 bp (PGL3-MCL-1(-200)) resulted in significant decreased transcriptional activity (Fig. 4d), suggesting a positive regulatory binding site between − 200 and − 400 bp. We therefore built the pGL3-basic-based luciferase reporter constructs with a mutated form of a putative binding element within this region (− 313 to − 308 bp), named as PGL3-MCL-1 (− 313 mut). Luciferase activity assay revealed a significantly lower transcriptional activity of PGL3-MCL-1 (− 313 mut) than wild-type PGL3-MCL-1 (− 1000) in response to XBP1s overexpression (Fig. 4e). The follow-up ChIP assay confirmed the direct binding element of XBP1s within the − 313 to − 308 bp region of the MCL-1 promoter (Fig. 4f). Therefore, the XBP1s could induce the MCL-1 expression by directly binding to the MCL-1 promoter.

As mentioned above, inhibition of MCL-1 expression induced the ER stress and the splicing of XBP1 (Fig. 3a), which indicated that MCL-1 might be feedback regulated by ER stress via XBP1. Thus, to explore the likelihood of the feedback regulation of MCL-1 transcription by XBP1, MSLC were transfected with XBP1-siRNA during tunicamycin treatment followed by evaluating MCL-1 expression. Results manifested that knockdown of XBP1 by siRNA inhibited the transcriptional response of MCL-1 even in the presence of tunicamycin (Fig. 4g), indicating that the upregulation of MCL-1 mRNA induced by ER stress is in a XBP1-dependent way. In addition, qPCR assay indicated that XBP1s overexpression significantly increase the expression of MCL-1 (Fig. 4c). Since XBP1 appears to have a major role in the regulation of MCL transcription more than the presence of ER stress, it follows that there exists a XBP1-ER stress-dependent regulation of MCL-1 transcription.

### Human MCL-1 was targeted by shrimp miR-965 in a cross-species manner

**Fig. 5:**
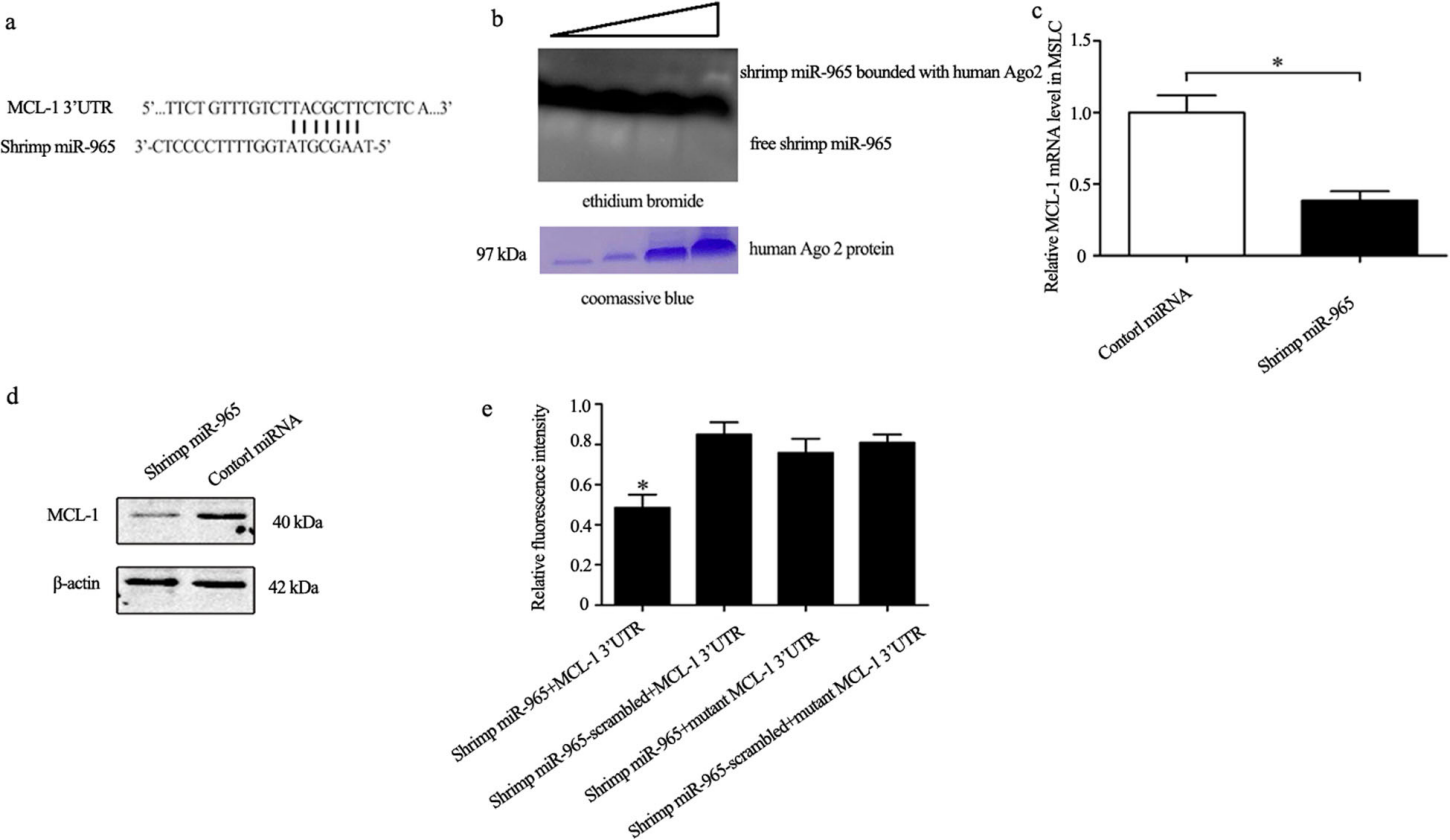
Human MCL-1 was targeted by shrimp miR-965 in a cross-species manner in MSLC. **a** Shrimp miR-965 was predicted to target human MCL-1 mRNA 3'UTR. **b** The interaction between shrimp miR-965 and human Ago2 protein. Shrimp miR-965 was incubated with recombinant human Ago2 protein. The mixture was separated by 1% agarose gel and stained with ethidium bromide to visualize the miRNA (top), followed by staining with Coomassie Blue (bottom). The wedges indicated the concentration gradient of the recombinant protein used. **c** The interaction between shrimp miR-965 and the MCL-1 mRNA in MSLC. Shrimp miR-965 or the control miRNA was transfected into MSLC. Twenty-four hours after transfection, the MCL-1 mRNA was detected using quantitative real-time PCR. **d** Western blot analysis of MCL-1 in shrimp miR-965-transfected MSLC. **e** The direct interaction between shrimp miR-965 and the MCL-1 gene. MSLC were co-transfected with shrimp miR-965 and a luciferase reporter fused with MCL-1 3′ UTR. At 24 h after transfection, the firefly and Renilla luciferase activities were analyzed. Control miRNA (shrimp miRNA-965-scrambled) and MCL-1 3′ UTR mutant were included in the co-transfections

To investigate whether miRNAs from food can be the drug source for melanoma therapy, shrimp miRNA candidates that targeted human MCL-1 were predicted. Based on miRNA sequence information, a few miRNAs from shrimp were predicted to target human MCL-1 3′ UTR. According to the prediction, shrimp miR-965 was one of those candidates as the seed sequence of shrimp miR-965 was complementary to the 3′ UTR of the MCL-1 gene (Fig. 5a). To test whether shrimp miR-965 targets human MCL-1, we firstly assessed the interaction between shrimp miR-965 and human Ago2 complex using EMSA. The results showed that the interaction of shrimp miR-965 with the human Ago2 yielded an RNA/protein shift band of expected mobility (Fig. 5b). This binding was robust because the binding band was increased with a higher Ago2 concentration. The result indicated that shrimp miR-965 could be loaded into the human Ago2 complex.

To further investigate whether human MCL-1 was a target of shrimp miR-965. Either shrimp miR-965 or the control miRNA was transfected into MSLC. As shown in Fig. 5c, d, MCL-1 expression was remarkably decreased in both mRNA level and protein level, indicating that MCL-1 was silenced by shrimp miR-965. We then performed a luciferase assay for MCL-1 and confirmed that the activity of wild-type MCL-1 was significantly reduced after the introduction of shrimp miR-965. On the other hand, a mutation in the MCL-1 3′ UTR binding site markedly abolished the ability of shrimp miR-965 to affect the luciferase activity (Fig. 5e). Taken together, these results revealed that shrimp miR-965 targeted human MCL-1 in MSLC in a cross-species manner.

### Shrimp miR-965 induced the MSLC ER stress, stemness loss, and apoptosis

**Fig. 6:**
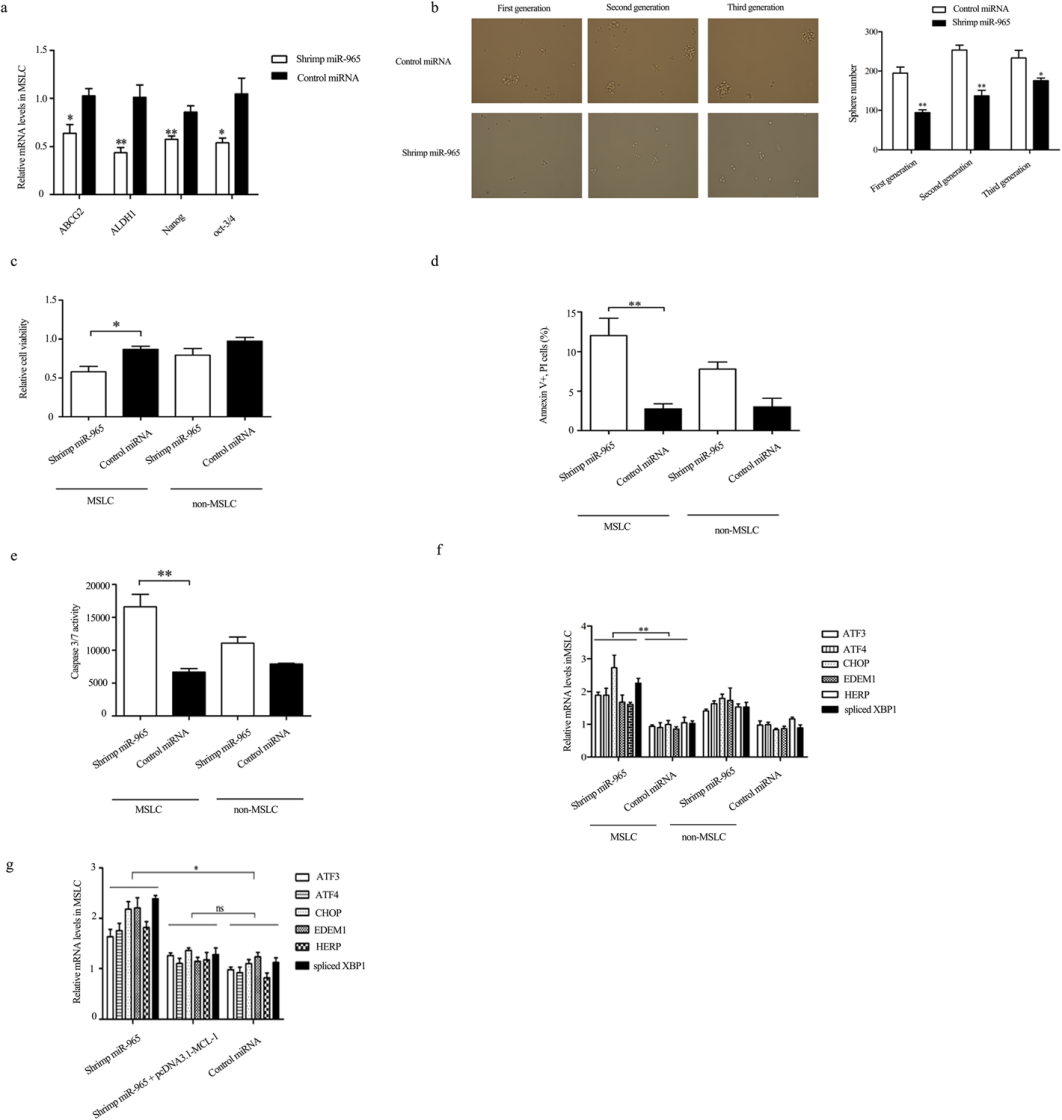
The influence of shrimp miR-965 on MSLC. **a** shrimp miR-965 inhibited the expression of cancer stemness-associated genes. MSLC were transfected with shrimp miR-965. At 24 h after transfection, the stemness-associated gene expression levels in MSLC were examined with quantitative real-time PCR. **b** Shrimp miR-965 reduced the tumorsphere formation capacity of MSLC. MSLC were transfected with shrimp miR-965 (or control) for 6 h followed by evaluating the tumorsphere formation capacity at consecutive generations. **c** Shrimp miR-965 reduced cell viability. The cell viability was evaluated at 24 h after transfection of MSLC and non-MSLC with shrimp miR-965. **d** The detection of apoptosis of shrimp miR-965-transfected cells using annexin V assays. Apoptosis of MSLC and non-MSLC was examined by flow cytometry at 24 h after the transfection of shrimp miR-965. **e** Shrimp miR-965 induced apoptosis of MSLC via caspase activity. The MSLC and non-MSLC at 24 h after the transfection of shrimp miR-965 were subjected to the caspase 3/7 activity analysis. **f** Shrimp miR-965 induced ER stress gene expression. MSLC or non-MSLC were transfected with shrimp miR-965. At 24 h after transfection, the ER stress gene expression levels in cells were examined with quantitative real-time PCR. In all panels, the statistical significance between treatments was indicated with stars (**p < 0.01)

To explore the function of shrimp miR-965 in MSLC, shrimp miR-965 was transfected into MSLC. The effects of shrimp miR-965 on the expression of cancer stemness-associated genes (ABCG2, ALDH1, Nanog, and Oct-3/4) were detected with qPCR. The results showed that shrimp miR-965 inhibited the expression of these genes significantly (Fig. 6a), indicating that shrimp miR-965 played a negative effect on the stemness properties of MSLC. The data from tumorsphere formation assays indicated that the sphere-forming ability of shrimp miR-965-transfected MSLC was inhibited compared with the control (Fig. 6b); what is more, 3 consecutive generations (transfected with shrimp miR-965) showed less tumorspheres when compared with the shrimp miR-965-scrambled-treated group, confirming suppressive effects of shrimp miR-965 on MSLC stemness. Taking together, these data strongly revealed that shrimp miR-965 played a negative role in regulating the stemness of MSLC. To reveal the shrimp miR-965-mediated inhibitory mechanism on tumorsphere formation of MSLC, the viability of shrimp miR-965-transfected MSLC was evaluated. According to the MTS assay data, the viability of shrimp miR-965-transfected MSLC was significantly suppressed compared with the control (Fig. 6c). However, shrimp miR-965 had no effect on the viability of non-MSLC (Fig. 6c).

The previous results associated with MCL-1 led us to further analyze the effects of shrimp miR-965 on cell apoptosis. The results showed that the apoptotic percentage of MSLC transfected with shrimp miR-965 was significantly increased compared with the control (Fig. 6d). But shrimp miR-965 had no significant effect on apoptosis of non-MSLC (Fig. 6d). The data indicated that shrimp miR-965 could promote apoptosis of MSLC. To investigate whether apoptosis induced by shrimp miR-965 was mediated through a caspase-dependent mechanism, we detected the activity of caspase3/7. As shown in Fig. 6e, the shrimp miR-965 transfection led to a significant increase of caspase 3/7 activity in MSLC but not in non-MSLC compared with the controls. These findings demonstrated that the downregulation of MCL-1 by shrimp miR-965 inhibited the proliferation of MSLC through a caspase-dependent apoptosis.

To further investigate the effects of shrimp miR-965 on ER stress of MSLC, we treated MSLC with shrimp miR-965 and detected ER stress-associated genes. As depicted in Fig. 6f, transfection of shrimp miR-965 increased the mRNA level of ER stress genes, including ATF3, ATF4, CHOP, EDEM1, HERP, and spliced XBP1. What is more, transfection of pcDNA3.1-MCL-1 blocked the induction of ER-associated genes (by shrimp miR-965) (Fig. 6g). Meanwhile, considering that knocked down MCL-1 induced ER stress (Fig. 3a), and MCL-1 was targeted by shrimp miR-965 (Fig. 5). Therefore, it could be reasonably concluded that shrimp miR-965 induced ER stress by silencing MCL-1.

Overall, shrimp miR-965 induced MSLC stemness loss, ER stress, and apoptosis.

### Shrimp miR-965 suppressed the tumor-initiating ability and tumor development of the MSLC in vivo

**Fig. 7:**
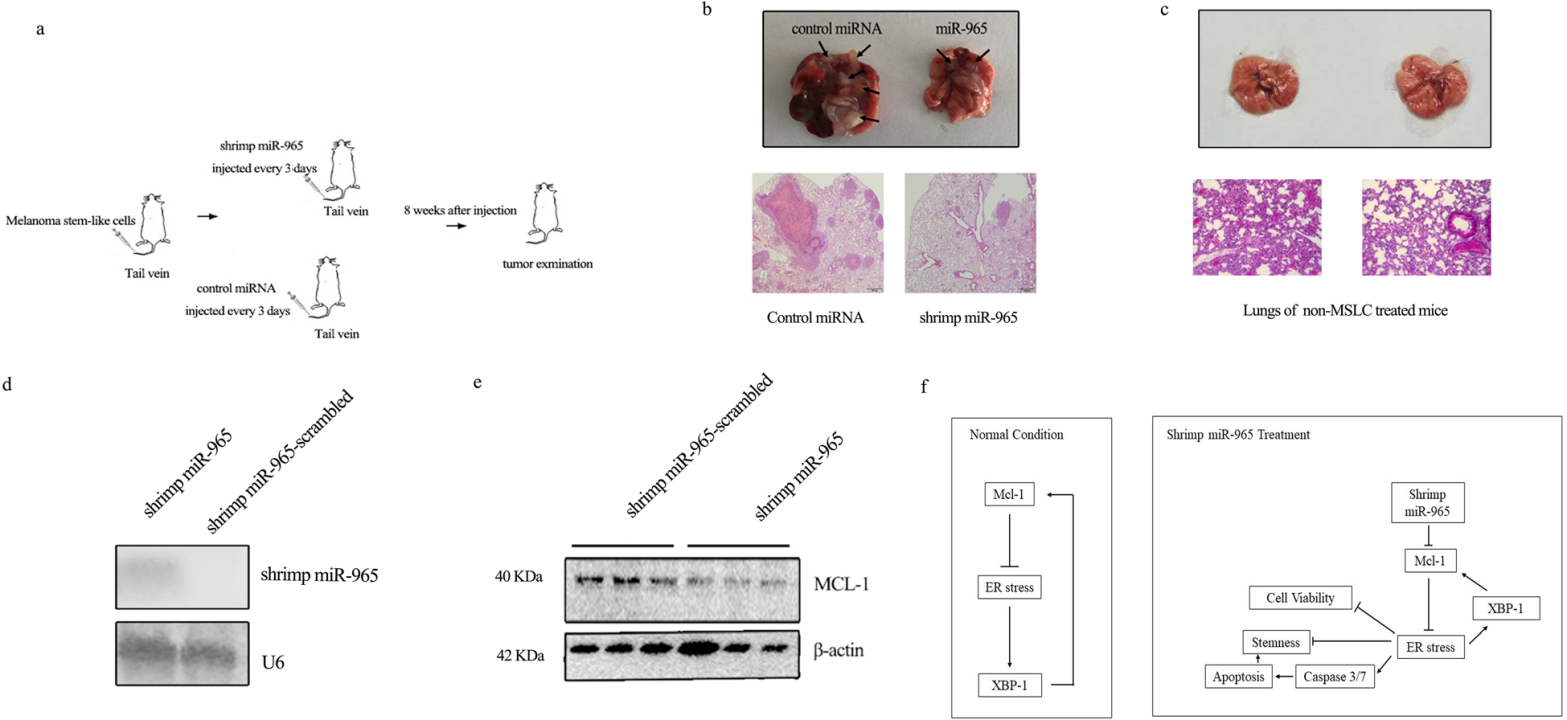
The effects of shrimp miR-965 on tumorigenesis of MSLC in vivo. **a** A flow diagram of the in vivo experiments. **b** The influence of shrimp miR-965 on tumors of lung tissue in mice. The MSLC were injected into nude mice, followed by treatment with shrimp miR-965 or shrimp miR-965-scrambled (control miRNA). Eight weeks after the first miRNA injection, the mice were sacrificed and the tumors of lung tissue were examined. The arrows indicated the tumors in lung tissues (upper panel). H&E staining for the lung biopsies of mice treated with shrimp miR-965 or shrimp miR-965-scrambled (control miRNA) (lower panel). **c** Mice were injected with non-MSLC for 8 weeks followed by examining the mouse lungs; upper panel indicated the mouse lungs, and the lower panel demonstrated the H&E staining results for lung biopsies from the non-MSLC-injected mice. **d** The detection of shrimp miR-965 in tumors of lung tissue. Northern blots were used to detect the levels of shrimp miR-965 in tumors in lung tissue of mice treated with shrimp miR-965 or control miRNA. **e** The detection of MCL expression. Western blots were used to detect protein levels of MCL-1 in tumors of mice treated with shrimp miR-965 or control miRNA. **f** The schematic presentation of shrimp miR-965 on the regulation of melanoma stem-like cell stemness. In normal condition (left panel), ER stress is negatively regulated by MCL-1; in detail, when ER stress is induced, XBP1 is spliced and then binds with MCL-1 promoter, thus activating the MCL-1 transcription; the increase of MCL-1 expression inhibits the ER stress in verse, which keeps the ER stress at a suitable level. When the shrimp miR-965 is introduced (right panel), MCL-1 is knocked down and the MCL-1-ER stress-XBP1 feedback loop is disrupted, which leads to the relief of ER stress inhibition and accumulation of ER stress. What is more, the accumulation of ER stress, on one hand, causes stemness loss, and on the other hand, enhances the Caspase 3/7 activity, thus triggering cell apoptosis. Meanwhile, cell viability decreased during ER stress accumulation

To explore the effect of shrimp miR-965 on the tumor-initiating ability and tumor development of MSLC in vivo, the nude mice were injected with MSLC from the tail vein, followed by the injection of shrimp miR-965 or control miRNA (Fig. 7a). Results manifested that the tumors sizes and tumor amount in the lungs of control miRNA-treated mice were larger than those in shrimp miR-965-treated mice (Fig. 7b), while non-MSLC did not generate any observable tumors in mice (Fig. 7c), showing that the shrimp miR-965 suppressed the tumor-initiating ability and tumor development of MSLC in vivo.

To further investigate whether the suppression by shrimp miR-965 in vivo was caused by the interaction between shrimp miR-965 and MCL-1, the expression levels of shrimp miR-965 and MCL-1 in solid tumors of mice treated with shrimp miR-965 or control miRNA were examined. The results of Northern blots indicated that shrimp miR-965 could be detected in tumors of shrimp miR-965-treated mice (Fig. 7d), indicating shrimp miR-965 was transferred into the tumors. Meanwhile, MCL-1 expression was significantly decreased in the tumors of shrimp miR-965-treated mice (Fig. 7e), showing that the targeting of MCL-1 by shrimp miR-965 led to the inhibition of the tumor-initiating ability and tumor development of MSLC in vivo.

Taken together, our results manifested that shrimp miR-965 suppressed the tumor-initiating ability and tumor development of MSLC in vivo by decreasing MCL-1 (Fig. 7f).

## Discussion

A variety of cancers including breast, pancreatic, head and neck, lung, ovarian, and melanoma have been shown to contain subpopulations of cells that exhibit stem-like characteristics [29–33]. Accumulating evidence suggests that CSCs may be largely responsible for the emergence of tumor resistance and a number of cell and animal studies strongly support the potential benefits of combining chemotherapeutic drugs with cancer stem cell-targeting agents [34]. ER stress has a dual impact on tumors. First, it has adaptive meaning, which promotes cellular repair and sustaining survival [35]. Second, over-activation of ER stress has cytotoxic effects on cancer cells, inducing apoptosis [35]. Therefore, strategies that cause overwhelming ER stress are reported to have a negative influence on cancer cells [36]. However, the role of ER stress in cancer stem-like cells has not been extensively researched. The myeloid cell leukemia sequence 1 (MCL-1), one of the key anti-apoptotic members of the B cell lymphoma-2 (Bcl-2) protein family, is currently under development as potential molecules target of chemotherapies, due to the role of MCL-1 in promoting survival as well as in conferring chemotherapeutic resistance of many types of cancers [37–39]. Similarly, the relationship between MCL-1 and MSLC is largely unknown. In this study, our results indicated that MCL-1 was an effective protein linking ER stress and stemness of MSLC. Silencing of MCL-1 resulted in ER stress as well as the significant decrease of stemness properties and cell viability in MSLC. Of note, the expression of MCL-1 was transcriptionally regulated by XBP1, which was a downstream factor of IRE1α ER-stress signaling branches. In addition, XBP1 had been reported to contribute to cancer progression with increased expression in many human cancers such as breast cancer, hepatocellular carcinoma, and pancreatic adenocarcinoma [40]. Our study revealed a significantly higher expression of MCL-1 in MSLC than non-MSLC, which indicated a specific role of MCL-1 in response to ER stress specially in MSLC. More importantly, the involvement of MCL-1 in the regulation of ER stress presents a feedback mechanism. Disrupting the balance of this feedback loop impairs the adaptive ability of MSLC to ER stress, the stemness properties, and the survival.

As it is well known, miRNA can induce the degradation or transcriptional inhibition of multiple seed sequence pairing mRNAs, with the aid of theAgo2 complex [41]. So, whether human mRNAs can be regulated by miRNAs from daily food. This has not been extensively investigated. In this study, we reported human MCL-1 could be regulated by a natural shrimp miRNA in a cross-species manner. According to our results, MCL-1 was a target of shrimp miR-965, further research manifested shrimp miR-965 could be loaded on human Ago2 protein and directly interacted with MCL-1 3′ UTR thus preventing the MSLC progression in vitro and in vivo. Therefore, our study indicated that natural miRNAs might be important sources for anti-tumor drugs.

## Conclusion

In summary, we indicated that XBP1 could regulate MCL-1 expression through direct binding to its promoter in response to ER stress; meanwhile, silencing of MCL-1 induced ER stress. Therefore, the MCL-1-ER stress-XBP1 feedback loop was revealed. More importantly, further results indicated that this feedback loop participated in the stemness regulation of MSLC. In addition, we found that a shrimp miR-965 could interact with the human Ago2 protein and suppressed the human MCL-1 expression by binding to the 3′ UTR of MCL-1 mRNA, thereby inhibiting the MSLC proliferation in vitro and in vivo in a cross-species manner. Our study suggested that MCL-1-ER stress-XBP1 feedback loop plays an important role in the regulation of MSLC viability, apoptosis, and stemness. We also found that shrimp miR-965, a natural food-derived miRNA, could suppress MSLC stemness by targeting MCL-1 and disrupting the balance of MCL-1-ER stress-XBP1 feedback loop. This study indicated an important mechanism of stemness regulation and demonstrated the significance of cross-species-derived miRNA as a promising natural drug in the treatment of melanoma.

## Data Availability

The datasets used and/or analyzed during the current study are available from the corresponding author on reasonable request.

## Abbreviations

ChIP: Chromatin immunoprecipitation
MCL-1: Myeloid cell leukemia sequence 1
MSLC: Melanoma stem-like cells
Non-MSLC: Melanoma non-stem-like cells
XBP1: X-box-binding protein 1
CSC: Cancer stem cells
FACS: Fluorescence-activated cell sorting
EMSA: Electrophoretic mobility shift ass

## References

[1] Silvers DN (1976). Focus on melanoma. J Dermatol Surg Oncol.

[2] Perez MC, Orcutt ST, Zager JS (2017). Current standards of surgical management in primary melanoma.

[3] Luke JJ (2017). Targeted agents and immunotherapies: optimizing outcomes in melanoma. Nat Rev Clin Oncol.

[4] Lonardo E, Hermann PC, Heeschen C (2010). Pancreatic cancer stem cells – update and future perspectives. Mol Oncol.

[5] Frosina G (2009). DNA repair and resistance of gliomas to chemotherapy and radiotherapy. Mol Cancer Res.

[6] Baumann M, Krause M, Hill R (2008). Exploring the role of cancer stem cells in radioresistance. Nat Rev Cancer.

[7] Hainaut P (2013). Targeting the hallmarks of cancer: towards a rational approach to next-generation cancer therapy. Curr Opin Oncol.

[8] Lagadec C (2009). TrkA overexpression enhances growth and metastasis of breast cancer cells. Oncogene.

[9] Brewster ME (2015). Preclinical and clinical studies.

[10] Krajewski S, et al. Immunohistochemical analysis of Mci-1 protein in human tissues. Am J Pathol. 1995;146:1309–19. PMC1870904 7778670

[11] Perciavalle RM, Opferman JT (2013). Delving deeper: MCL-1’s contributions to normal and cancer biology. Trends Cell Biol.

[12] Annis MG (2004). There is more to life and death than mitochondria: Bcl-2 proteins at the endoplasmic reticulum. Biochim Biophys Acta.

[13] Akgul C (2009). Mcl-1 is a potential therapeutic target in multiple types of cancer. Cell Mol Life Sci.

[14] Shamas-Din A (2011). BH3-only proteins: orchestrators of apoptosis. Biochim Biophys Acta.

[15] D’Alessio M (2005). Oxidative Bax dimerization promotes its translocation to mitochondria independently of apoptosis. FASEB J.

[16] Placzek WJ (2010). A survey of the anti-apoptotic Bcl-2 subfamily expression in cancer types provides a platform to predict the efficacy of Bcl-2 antagonists in cancer therapy. Cell Death Dis.

[17] Cai Y (2009). A brief review on the mechanisms of miRNA regulation. Genomics Proteomics Bioinformatics.

[18] Shimono Y (2009). Downregulation of miRNA-200c links breast cancer stem cells with normal stem cells. Cell.

[19] Yang F (2017). Shrimp miR-S8 suppresses the stemness of human melanoma stem-like cells by targeting the transcription factor YB-1. Cancer Res.

[20] Céline G, Eileen W (2005). BH3-only proteins in control: specificity regulates MCL-1 and BAK-mediated apoptosis. Genes Dev.

[21] Yuchun Luo NN, Fujita M (2013). Isolation of human melanoma stem cells using ALDH as a marker. Curr Protoc Stem Cell Biol.

[22] Karmakar S (2016). Abstract 2495: hPaf1/PD2 interacts with OCT3/4 in maintenance of the self-renewal process of ovarian cancer stem cells. Cancer Res.

[23] Ding XW, Wu J-h, Jiang C-p (2010). ABCG2: a potential marker of stem cells and novel target in stem cell and cancer therapy. Life Sci.

[24] Wang ML, Chiou SH, Wu CW (2013). Targeting cancer stem cells: emerging role of Nanog transcription factor. Onco Targets Ther.

[25] Chen CJ (2010). Abstract 1198: adaptation to ER stress as a driver of increased expression of Mcl-1 with melanoma progression. Cancer Res.

[26] Lin J, et al. Regulation of cancer stem cell self-renewal by HOXB9 antagonizes ER stress-induced melanoma cell apoptosis via the miR-765-FOXA2 axis. J Invest Dermatol. 2018;138(7):1609–19. 10.1016/j.jid.2018.01.023 29408459

[27] Chang CW (2018). ROS-independent ER stress-mediated NRF2 activation promotes Warburg effect to maintain stemness-associated properties of cancer-initiating cells. Cell Death Dis.

[28] Yoshida H (2001). XBP1 mRNA is induced by ATF6 and spliced by IRE1 in response to ER stress to produce a highly active transcription factor. Cell.

[29] Gomez-Cabrero A, Wrasidlo W, Reisfeld RA (2013). IMD-0354 targets breast cancer stem cells: a novel approach for an adjuvant to chemotherapy to prevent multidrug resistance in a murine model. PLoS One.

[30] Liu P (2013). Disulfiram targets cancer stem-like cells and reverses resistance and cross-resistance in acquired paclitaxel-resistant triple-negative breast cancer cells. Br J Cancer.

[31] Lim YC (2011). Cancer stem cell traits in squamospheres derived from primary head and neck squamous cell carcinomas. Oral Oncol.

[32] Galli R (2004). Isolation and characterization of tumorigenic, stem-like neural precursors from human glioblastoma. Cancer Res.

[33] Sandercock J (2002). First-line treatment for advanced ovarian cancer: paclitaxel, platinum and the evidence. Br J Cancer.

[34] Yi L (2012). A small-molecule inhibitor of glucose transporter 1 downregulates glycolysis, induces cell-cycle arrest, and inhibits cancer cell growth in vitro and in vivo. Mol Cancer Ther.

[35] Puthalakath H (2007). ER stress triggers apoptosis by activating BH3-only protein Bim. Cell.

[36] Verfaillie T, Garg AD, Agostinis P (2013). Targeting ER stress induced apoptosis and inflammation in cancer. Cancer Lett.

[37] Gores GJ, Kaufmann SH (2012). Selectively targeting Mcl-1 for the treatment of acute myelogenous leukemia and solid tumors. Genes Dev.

[38] Fan F (2014). Targeting Mcl-1 for multiple myeloma (MM) therapy: drug-induced generation of Mcl-1 fragment Mcl-1128–350 triggers MM cell death via c-Jun upregulation. Cancer Lett.

[39] Zangemeister-Wittke U, Huwiler A (2006). Antisense targeting of Mcl-1 has therapeutic potential in gastric cancer. Cancer Biol Ther.

[40] Koong AC, Chauhan V, Romero-Ramirez L (2006). Targeting XBP-1 as a novel anti-cancer strategy. Cancer Biol Ther.

[41] Bartel DP (2009). MicroRNAs: target recognition and regulatory functions. Cell.

